# Transcriptome Analysis of Postharvest *Lentinula edodes* Cell Wall Metabolism During Storage Indicating a Laccase-Mediated Regulatory Network

**DOI:** 10.3390/foods15061039

**Published:** 2026-03-16

**Authors:** Yuan Gao, Qimeng Liang, Yanyan Liu, Tinging Ma, Ziwei Hou, Hongxu Zhu, Jun Huang

**Affiliations:** School of Biological and Chemical Engineering, Zhejiang University of Science and Technology, Hangzhou 310023, China

**Keywords:** shiitake mushroom, quality deterioration, oxidative stress, cell wall remodeling, laccase

## Abstract

Postharvest *Lentinula edodes* (shiitake mushroom) undergoes rapid textural deterioration, which is primarily driven by complex cell wall remodeling. This study investigates the physiological and transcriptomic changes in *L. edodes* during storage at 4 °C for 8 days. Results showed that cellulose content significantly decreased, while chitin and β-glucan levels exhibited anomalous increases, accompanied by a surge in the activities of cellulase, chitinase, and β-1,3-glucanase. Concurrently, intensifying membrane lipid peroxidation and an imbalance in reactive oxygen species (ROS) homeostasis were observed. Transcriptomic analysis identified 2204 and 1808 differentially expressed genes (DEGs) at the middle (4 d) and late (8 d) storage stages, respectively. Partial Least Squares Regression (PLSR) identified a core module of nine key regulatory genes (VIP > 1.0), including β-glucanase, laccase, and catalase, which significantly contributed to the physiological shifts. The results suggest that an upstream ROS imbalance may contribute to the dysregulation of midstream laccases, potentially reducing the oxidative cross-linking of phenolic components and loosening the cell wall matrix. These alterations may increase the accessibility of structural polysaccharides to downstream cell wall-degrading enzymes, which could contribute to structural collapse, although functional validation is required to establish causality. These findings provide a gene-level framework for understanding postharvest edible fungi physiology.

## 1. Introduction

*Lentinula edodes* (*L. edodes*), commonly known as shiitake, is a traditional edible fungus renowned for its characteristic flavor and diverse bioactivities [1]. Originating in China, it has been consumed and used medicinally for millennia and remains widely popular throughout East and Southeast Asia [2]. *L. edodes* is rich in various essential nutrients and bioactive compounds, including polyphenols, polysaccharides, and antioxidants [3]. In vitro and in vivo studies consistently confirm that these components provide its fruiting bodies and mycelium with multiple beneficial health effects on the human body [4]. Postharvest increases in transpiration, respiration, and energy metabolism promote textural deterioration [5]. As a result, mushrooms soften, discolor, develop off-flavors, and lose nutrients, degrading quality and shortening shelf life.

Postharvest autolysis and softening in edible fungi, fruits, and vegetables are manifested as reductions in firmness and changes in texture and are commonly associated with disruptions in cell-wall metabolism, including structural alterations of the cell wall and decreased cell turgor [6]. In fruits and vegetables, this process is well-documented and primarily involves the enzymatic disassembly of the plant cell wall, a complex network of cellulose, hemicellulose, and pectin [7,8]. For fleshy fruits, softening is primarily the result of programmed modification of the primary cell wall and the middle lamella. Pectin de-esterification, depolymerization, and solubilization mediated by pectin methyl-esterase (PME), pectate lyase (PL), and polygalacturonase (PG), together with hemicellulose remodeling and local alterations in cellulose–matrix interactions, weaken cell-to-cell adhesion and compromise tissue integrity. These changes lead to a characteristic loss of firmness and an increased susceptibility to mechanical damage and pathogen invasion [9]. Leafy vegetables undergo a related but much faster decline in quality because their high surface-to-volume ratio and large water content accelerate respiration, transpiration, and enzymatic activity, and cell-wall–modifying and oxidative enzymes rapidly reduce turgor and textural quality [9,10].

However, the mechanism of textural degradation in edible fungi is fundamentally different due to its unique cell wall architecture. Fungal cell walls lack pectin and are instead a dynamic matrix of glycoproteins and polysaccharides, primarily chitin, β-glucans, and cellulose [11]. Recent studies show that firmness loss in *L. edodes* strongly correlates with increased expression and activity of endogenous hydrolytic enzymes, especially β-1,3-glucanases and chitinases [12]. After harvest, upregulation of glucanases, chitinases, and cellulases progressively removes these polysaccharides, which leads to tissue collapse and softening [13]. This degradation also reduces soluble nutrients and the volatile compounds that contribute to mushroom flavor [13].

Empirical studies (e.g., equilibrium modified-atmosphere packaging) have shown that controlling gas composition, humidity, and mechanical damage can slow the activity of wall-degrading enzymes and preserve higher levels of wall polymers and volatile/flavor compounds during storage [14,15]. Because cell-wall remodeling is both enzymatic and transcriptionally regulated, transcriptomic analyses have become essential tools to identify the suites of hydrolases, lytic enzymes, and upstream transcription factors that govern postharvest wall disassembly in different commodities. Such knowledge underpins targeted interventions to retard wall degradation and extend marketable shelf life [5,6]. However, few studies have employed transcriptomic analysis to investigate postharvest cell-wall metabolism in *L. edodes*. This study aims to identify transcripts and pathways involved in cell-wall metabolism in postharvest *L. edodes* fruiting bodies using transcriptomic analysis and seeks to clarify the molecular mechanisms underlying postharvest cell-wall remodeling. The results will provide new insights into the physiology of postharvest edible fungi.

## 2. Materials and Methods

### 2.1. Mushroom Materials and Treatment

Fresh *L. edodes* (No. f6) used in this study was purchased from Wuyi Innovative Edible Fungi Co., Ltd. (Jinhua, China), and transported to the laboratory within 4 h after harvest. *L. edodes* fruiting bodies of uniform size and free of blemishes were selected. Samples were randomly assigned to groups of equal weight (500 ± 10 g per group). *L. edodes* were stored at 4 ± 1 °C and 85 ± 5% relative humidity for 8 days. Sampling was performed every 2 days. All collected samples were frozen in liquid nitrogen and stored at −80 °C.

### 2.2. Determination of Cellulose, Chitin, and Glucan Contents

The chitin content in *L. edodes* fruiting bodies was measured using an ELISA assay kit (Kejing Biological Technology, Yancheng, China). The content of cellulose in *L. edodes* fruiting bodies was measured based on the previous method of Yang [16] with slight modifications. Samples (0.05 g, air-dried) were placed in a 50 mL volumetric flask, cooled in an ice bath, and treated with 30–35 mL cold 60% H_2_SO_4_ for 30 min. The digest was then diluted to volume with 60% H_2_SO_4_, mixed, and filtered through a Buchner funnel. A 5.0 mL aliquot of the filtrate was transferred to a new 50 mL volumetric flask, kept in an ice bath, diluted to volume with distilled water, mixed, and filtered. Two milliliters of the resulting solution were mixed with 0.5 mL of 2% anthrone reagent in a test tube, and 5.0 mL of concentrated H_2_SO_4_ was carefully added along the tube wall; after gentle inversion to promote hydrolysis and the appearance of anthrone floccules, the tube was shaken vigorously to dissolve them, heated in a boiling-water bath for 10 min, cooled, and measured at 620 nm. Quantification was performed using a microplate reader (Multiskan GO, Thermo Fisher Scientific, Waltham, MA, USA). The content of glucan in *L. edodes* fruiting bodies was measured using a commercial detection assay (Megazyme International, Bray, Ireland). All cell wall polysaccharide contents were expressed on a dry weight (DW) basis to minimize the influence of water loss during storage.

### 2.3. Evaluation of Cellulase, Chitinase, and Glucanase Activities

The activities of cellulase (Nanjing Jiancheng Bioengineering Institute, China, A138-1-1), chitinase (Nanjing Jiancheng Bioengineering Institute, Nanjing, China, A139-1-1), and β-1,3-glucanase (Yuanye Bio-Technology, Shanghai, China, R41759) were measured using a commercial detection assay kit. Quantification was performed using a microplate reader (Multiskan GO, Thermo Fisher Scientific, Waltham, MA, USA).

### 2.4. Determination of Antioxidant Enzyme Activities and Membrane Lipid Peroxidation

The activities of lipoxygenase (LOX, Solarbio Life Science, Beijing, China, BC0320), superoxide dismutase (SOD, Nanjing Jiancheng Bioengineering Institute, Nanjing, China, A001-3-2), peroxidase (POD, Nanjing Jiancheng Bioengineering Institute, Nanjing, China, A084-3-1), catalase (CAT, Nanjing Jiancheng Bioengineering Institute, Nanjing, China, A007-1-1), polyphenol oxidase (PPO, Nanjing Jiancheng Bioengineering Institute, Nanjing, China, A136-1-1) activities and malondialdehyde (MDA, Nanjing Jiancheng Bioengineering Institute, Nanjing, China, A003-1-2) content were determined by using commercial detection assay kits (Nanjing Jiancheng Bioengineering Institute, Nanjing, China). Quantification was performed using a microplate reader (Multiskan GO, Thermo Fisher Scientific, Waltham, MA, USA).

### 2.5. RNA-Seq Sequencing

#### 2.5.1. RNA Extraction

For transcriptome analysis, three biological replicates were prepared at the beginning (T0, Day 0), middle (T1, Day 4), and end (T2, Day 8). For clarity throughout the manuscript and Supplementary Material, we use the following notation: T0 = Day 0 (fresh control), T1 = Day 4 (intermediate/transition stage), and T2 = Day 8 (late-stage deterioration). Total RNA was isolated from L. edodes tissues using TRIzol^®^ Reagent (Invitrogen, Thermo Fisher Scientific, Waltham, MA, USA) following the manufacturer’s protocol, and residual genomic DNA was removed with DNase I (TaKaRa, Shiga, Kusatsu, Japan). RNA integrity was assessed using an Agilent 2100 Bioanalyzer (Agilent Technologies, Santa Clara, CA, USA), and RNA concentration was measured with a NanoDrop ND-2000 spectrophotometer (Thermo Fisher Scientific, Waltham, MA, USA). Samples meeting the following quality criteria (OD_260_/_280_ = 1.8–2.2, OD_260_/_230_ ≥ 2.0, RIN ≥ 6.5, 28S:18S ≥ 1.0, and total RNA > 10 μg) were selected for sequencing library construction.

#### 2.5.2. Library Preparation, and Illumina Hiseq Sequencing

RNA-seq transcriptome libraries were constructed using the TruSeq™ RNA Sample Preparation Kit (Illumina, San Diego, CA, USA) with 1 μg of total RNA as input. Briefly, mRNA was enriched by poly (A) selection using oligo (dT) magnetic beads and subsequently fragmented in fragmentation buffer. First- and second-strand cDNA synthesis, end repair, A-tailing, and ligation of Illumina indexed adapters were carried out in accordance with the manufacturer’s instructions. The resulting libraries were size-selected on 2% Low Range Ultra agarose gels to obtain cDNA fragments of 200–300 bp, followed by PCR amplification for 15 cycles using Phusion High-Fidelity DNA polymerase (New England Biolabs, Ipswich, MA, USA). Library concentrations were determined using a TBS380 fluorometer, and paired-end sequencing (150 bp × 2) was performed on an Illumina NovaSeq 6000 platform (Illumina, San Diego, CA, USA).

#### 2.5.3. Reads Quality Control and Mapping

Raw paired-end reads were filtered and quality-trimmed using Trimmomatic v0.36 with the parameters SLIDINGWINDOW:4:15 and MINLEN:75. The resulting high-quality reads were then independently aligned to the *L. edodes* reference genome (Genome assembly Lenedo1, GCF_021015755.1) in a strand-specific manner using HISAT2 with default settings. Alignment quality and overall sequencing performance were assessed with Qualimap v2.2.1. Finally, gene-level read counts were generated using HTSeq (v0.11.1).

#### 2.5.4. Differential Expression Analysis and Functional Enrichment

To identify differentially expressed genes (DEGs) between the two sample groups, gene expression levels were quantified using the fragments per kilobase of exon per million mapped reads (FPKM) method. Differential expression analysis was performed with the R package edgeRv3.42.4. Genes with an absolute log_2_ fold change > 1 (|log_2_FC| > 1) and a false discovery rate (FDR) < 0.05 were considered significantly differentially expressed. Functional annotation and enrichment analyses of DEGs were conducted using GOATOOLS for Gene Ontology (GO) enrichment and KOBAS for Kyoto Encyclopedia of Genes and Genomes (KEGG) pathway analysis. GO terms and KEGG pathways with a Bonferroni-corrected *p* value < 0.05 were regarded as significantly enriched.

### 2.6. Statistical Analysis

All experiments were performed using three independent biological replicates per time point (*n* = 3). All experiments were conducted in triplicate, and results are presented as the mean ± standard deviation. Statistical analyses were performed using one-way analysis of variance (ANOVA) with SPSS softwarev22.0. Differences were considered statistically significant at *p* < 0.05 based on Duncan’s multiple range test.

The relationships between mushroom quality parameters and the differential expression of DEGs were further evaluated using partial least squares regression (PLSR). All statistical analyses were performed in R (version 4.2.3; R Foundation for Statistical Computing, Vienna, Austria). Partial least squares regression (PLSR) linking gene expression (predictor variables) and physiological indicators (response variables) was conducted using the plsr function from the pls package (v2.8-1) with preprocessing and model selection implemented via the caret package (v6.0-92). Prior to modeling, predictor and response variables were autoscaled (mean-centered and divided by the standard deviation). The number of latent components was determined by repeated 10-fold cross-validation (10 folds × 10 repeats) using root mean squared error of prediction (RMSEP) as the selection criterion; the final model used three latent components, which minimized RMSEP and satisfied the one-standard-error rule. Model performance was assessed by cumulative explained variances R^2^X and R^2^Y, cross-validated predictive ability Q^2^, and RMSEP. Variable importance was evaluated using the Variable Importance in Projection (VIP) metric; variables with VIP > 1.0 were considered important. To avoid overfitting, we set a fixed random seed for reproducibility, used repeated cross-validation, limited the number of latent components by the CV rule above, and performed permutation testing (*n* = 1000 permutations) to assess whether model Q^2^ exceeded values expected by chance. VIP stability was further evaluated via bootstrap resampling (*n* = 500).

## 3. Results

### 3.1. Quality Deterioration of L. edodes During Storage

**Figure 1:**
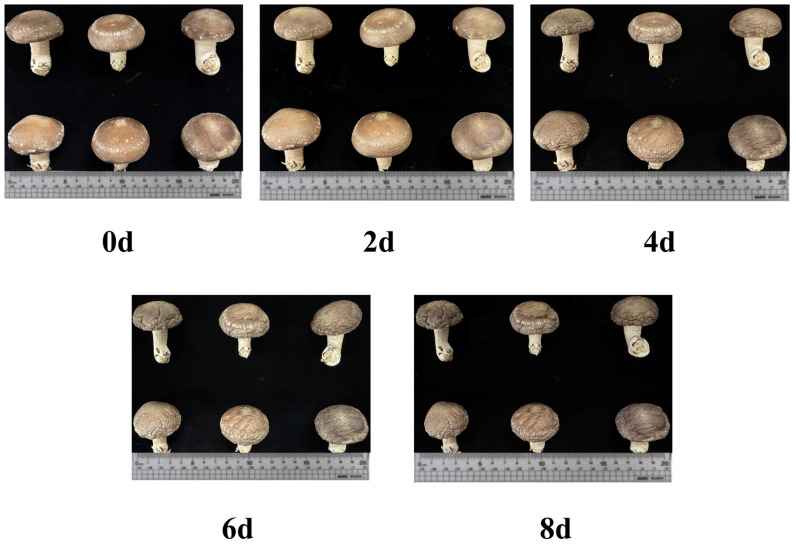
Appearance changes in *Lentinula edodes* fruiting bodies during postharvest storage at 4 °C.

Significant changes occurred in the fruiting bodies of *L. edodes* during storage (Figure 1). During the initial 0–2 days, the fruiting bodies remained firm and turgid, exhibiting a characteristic light-brown pileus with densely arranged white lamellae. With the progression of storage time, visual quality continuously deteriorated through pileus expansion accompanied by partial veil rupture, distinct browning, and a wrinkled, desiccated surface, which resulted in a general decline in external appearance.

### 3.2. Changes in Cellulose, Chitin, and Glucan Contents and Related Metabolism Enzyme Activities During Storage

**Figure 2:**
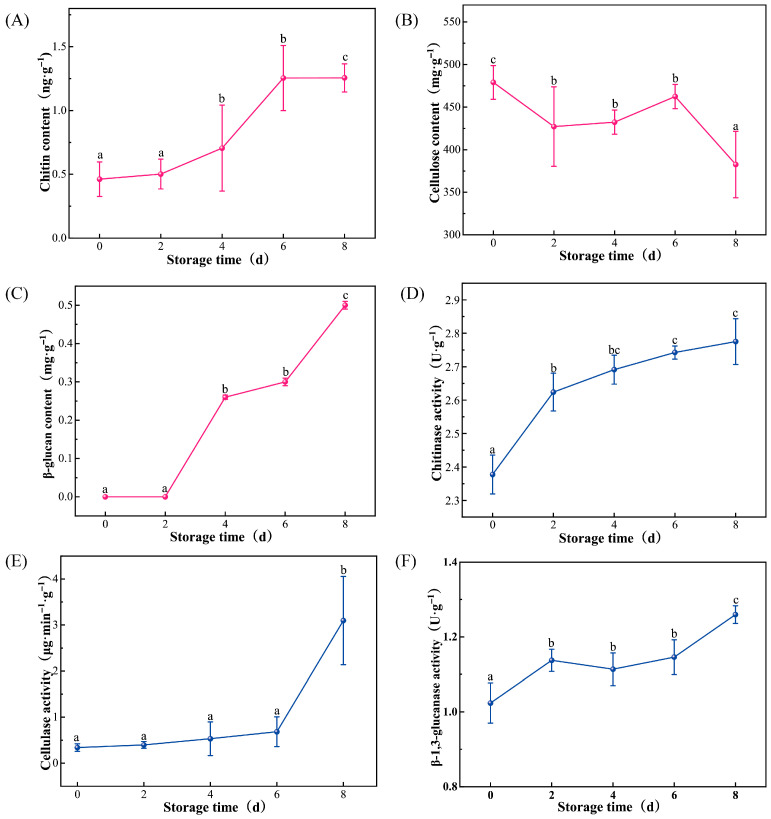
Changes in chitin (**A**), cellulose (**B**), glucan (**C**) contents, and chitinase (**D**), cellulase (**E**), and β-1,3-glucanase activity (**F**) in *Lentinula edodes* during postharvest storage. Each data point is the mean ± standard error of three biological repetitions. Different letters (a–c) indicate significant differences (*p* < 0.05) between groups. All cell wall polysaccharide contents were expressed on a dry weight basis.

During the 8 day storage period, the contents of chitin, cellulose, and β-glucan in *L. edodes* exhibited distinct trends (Figure 2A–C). The chitin content increased significantly with storage time (0.46 ng/g at Day 0) and was followed by a gradual rise from 2 d to Day 4 with no significant difference (*p* > 0.05). From 6 d, the chitin content surged significantly to 1.25 ng/g and reached a peak of 2.13 ng/g at Day 8, which was significantly higher than all other groups (*p* < 0.05). In contrast, the cellulose content showed an overall downward trend during storage, which was highest on Day 0 at 479.01 mg/g. Although slight fluctuations were observed from 2 d to 6 d (427.14 to 462.41 mg/g), the levels remained significantly lower than the initial value (*p* < 0.05). By Day 8, the cellulose content had significantly dropped to a minimum of 382.63 mg/g. β-glucan was not detected during the first 2 days of storage. It was first detected on Day 4, remained relatively stable until 6 d (*p* > 0.05), and increased significantly to 0.50 mg/g on Day 8 (*p* < 0.05).

Corresponding to the changes in cell wall components, the activities of the three degrading enzymes showed varying degrees of enhancement during storage (Figure 2D–F). Chitinase activity rose steadily from 2.38 U/g on Day 0 and increased significantly to 2.62 U/g on 2 d. The activities on Day 6 and Day 8 reached 2.74 U/g and 2.78 U/g, respectively, with no significant difference between the two (*p* > 0.05), though both were significantly higher than at the beginning of storage (*p* < 0.05). Cellulase activity remained at a low level with no significant difference during the first 6 days (*p* > 0.05), ranging from 0.34 to 0.6 U/g. However, on Day 8, cellulase activity surged significantly to 3.09 U/g, representing an approximately 9-fold increase compared to the initial activity (*p* < 0.05). β-1,3-glucanase activity showed a fluctuating upward trend. The activity was 1.02 U/g on Day 0. While 2 d, Day 4, and 6 d showed significantly higher activities than Day 0 (*p* < 0.05), no significant differences were observed among these three groups (*p* > 0.05). By Day 8, the enzyme activity reached a maximum of 1.26 U/g, which was significantly higher than during other storage stages (*p* < 0.05).

### 3.3. Changes in Membrane Lipid Peroxidation and Antioxidant Enzyme Activities During Storage

**Figure 3:**
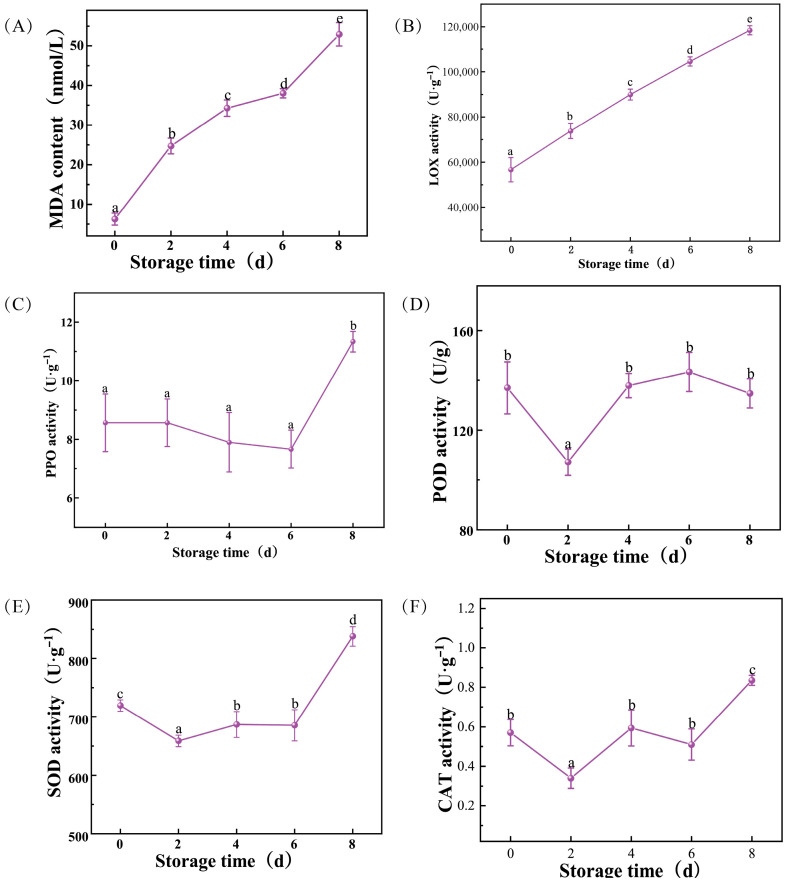
Changes in MDA content (**A**), and LOX (**B**), PPO (**C**), POD (**D**), SOD (**E**), and CAT (**F**) activities in *Lentinula edodes* during postharvest storage. Each data point is the mean ± standard error of three biological repetitions. Different letters (a–e) indicate significant differences (*p* < 0.05) between groups.

During the 8 day storage period, the degree of membrane lipid peroxidation in *L. edodes* fruiting bodies intensified significantly with storage time (Figure 3A,B). The MDA content showed a continuous and significant upward trend. The lowest MDA content was observed on Day 0 (6.26 nmol/g). Subsequently, this indicator showed significant differences among all storage intervals (*p* < 0.05), reaching a peak of 52.94 nmol/g on Day 8, which was approximately 7.5 times higher than the initial level. Consistent with the trend of MDA, LOX activity also increased significantly with storage time. LOX activity rose continuously from 56,666.67 U/g on Day 0, with significant differences observed between each storage time point (*p* < 0.05). By Day 8, LOX activity reached a maximum of 118,466.67 U/g, indicating severe oxidative degradation of membrane lipids in the late stage of storage.

The activities of related enzymes exhibited different fluctuation patterns during storage (Figure 3C–F). PPO activity remained relatively stable during the first 6 days of storage with no significant differences (*p* > 0.05). However, on Day 8, PPO activity increased significantly to 11.33 U/g (*p* < 0.05). POD activity showed a trend of initial decrease followed by an increase. The initial activity was 137.0 U/g on Day 0, which decreased significantly to 107.17 U/g on 2 d (*p* < 0.05). Subsequently, from Day 4 to Day 8, POD activity recovered, all showing no significant difference from the initial level. CAT and SOD activities showed a fluctuating trend. (Figure 3E,F). CAT activity was decreased significantly from Day 0 to 2 d (*p* < 0.05). Subsequently, on Day 4 and 6 d, CAT activity was recovered, showing no significant difference from Day 0. On Day 8, the activity increased significantly to a maximum of 0.84 U/g, which was significantly higher than the initial value (*p* < 0.05). SOD activity decreased significantly and remained at a lower level, ranging from 658.80 to 686.88 U/g, compared to Day 0 (*p* < 0.05). By Day 8, the enzyme activity surged to a peak of 837.6 U/g, higher than on other days (*p* < 0.05).

Based on observed physiological trends, Day 0, Day 4 and Day 8 were selected for transcriptomic sequencing to represent baseline (T0, Day 0), a critical transition (T1, Day 4) and late-stage deterioration (T2, Day 8), respectively. T0 served as the fresh control, representing the initial physiological and biochemical state of *L. edodes*. T1 represented a transition stage, characterized by the onset of compositional and enzymatic changes, including the first detection of β-glucan (0.26 mg·g^−1^) and increased chitinase activity. T2 corresponded to the advanced storage stage, showing maximal alterations such as peak membrane lipid peroxidation (MDA and LOX), elevated cellulase activity and chitin levels, and pronounced changes in PPO and SOD activities.

### 3.4. RNA-Seq Analysis and Identification of DEGs

#### 3.4.1. Transcripts Identification and Differential Expression Analysis

After adapter trimming and quality filtering, samples retained 45,672,463.11 ± 2,465,164.93 clean reads per sample, with an average Q30 of 97.98%. Alignment to the *L. edodes* reference genome resulted in an average mapping rate of 85.78%. These QC metrics indicate high-quality data suitable for downstream differential expression analysis. RNA-seq was employed to evaluate transcriptomic regulation in postharvest *L. edodes* fruiting bodies, with a total of 12,007 genes identified. Principal component analysis (PCA) and hierarchical cluster analysis demonstrate high biological reproducibility, significant temporal transcriptomic shifts, and highly characteristic gene expression profiles of *L. edodes* across different storage stages, which collectively reflect the dynamic evolution of its physiological state (Figure S1). Transcripts with |log Fold Change (FC)| ≥ 1 and FDR ≤ 0.05 were defined as DEGs (Table S1), and volcano plots intuitively visualized the differential expression patterns of these DEGs (Figure S2). A total of 2204 DEGs were identified in the 4-day storage group compared with the control group (T1/T0 comparison), of which 1292 DEGs were up-regulated, and 912 DEGs were down-regulated (Figure S2, Table S1). A total of 1808 DEGs were identified during the 8-day storage period (T2/T0 comparison), of which 1106 were up-regulated and 702 were down-regulated (Figure S2). Whereas in the T2/T1 group, 291 of the 617 DEGs were up-regulated and 326 were down-regulated (Figure S2).

#### 3.4.2. Enrichment Analysis of DEGs

To annotate the biological functions of all identified DEGs, functional classification and enrichment analyses were performed based on the GO and KEGG databases. Based on the GO enrichment bubble plot, the most significantly enriched DEGs in the T1/T0 comparison were associated with response to salt stress, protein self-association, response to hydrogen peroxide, response to reactive oxygen species, and 2-oxoglutarate-dependent dioxygenase activity (Figure S2A). The T2/T0 comparison shared similar enriched GO terms with the T1/T0 comparison, including response to salt stress, protein self-association, response to hydrogen peroxide, and 2-oxoglutarate-dependent dioxygenase activity (Figure S2B). By contrast, DEGs in the T2/T1 comparison were predominantly enriched in cell wall metabolism-related terms (Figure S2C), namely external encapsulating structure, cell wall, fungal-type cell wall, and structural constituent of cell wall, which was consistent with our previous physiological data demonstrating that marked changes in cell wall components and metabolic enzyme activities occurred on the 8th day of storage. For KEGG pathway enrichment analysis, the most significantly enriched DEGs in the T1/T0 comparison were associated with microbial metabolism in diverse environments, other glycan degradation, glycolysis/gluconeogenesis, and glyoxylate and dicarboxylate metabolism (Figure S2D). DEGs in the T2/T0 comparison were predominantly enriched in steroid biosynthesis, carbon fixation in photosynthetic organisms, and carbon metabolism (Figure S2E). By contrast, DEGs in the T2/T1 comparison were only enriched in steroid biosynthesis (Figure S2F).

#### 3.4.3. Transcriptomic Analysis of DEGs Related to Cell Wall Metabolism and Oxidative Stress

By screening the transcriptome data of *L. edodes* across different storage stages (T0, T1, and T2), multiple DEGs associated with cell wall metabolism were identified. These DEGs are primarily involved in the metabolism of chitin, glucan, and cellulose, as well as lignin-related modification processes (Table 1).

Regarding degradation-related genes, chitinase and glucanase displayed significant dynamic alterations. During the T1/T0 comparison, genes encoding β-glucosidase (C8R40DRAFT_1102963 and C8R40DRAFT_1173381) and cellulase CEL7A (C8R40DRAFT_1127624) were significantly up-regulated. As the storage period extended, the expression level of cellulase CEL6B (C8R40DRAFT_1075571) continued to increase, reaching a log_2_FC of 1.45 in the T2/T0 comparison. The genes encoding glucanase (C8R40DRAFT_774955 and C8R40DRAFT_775537) were significantly up-regulated during different periods. For genes involved in synthesis and modification, the chitin-binding domain-containing protein (C8R40DRAFT_229556) was significantly down-regulated across the entire storage period, with its log_2_FC increasing to −1.14 in the T1/T0 comparison.

The expression profiles of the laccase family genes exhibited distinct member-specific responses. In the early storage stage (T1/T0 comparison), several laccase genes (C8R40DRAFT_642440, C8R40DRAFT_1068383) showed a significant down-regulation pattern. Specifically, laccase 1 (C8R40DRAFT_1025595) exhibited marked differential expression, showing a consistent upward trend alongside other members. The down-regulation trend of laccases (C8R40DRAFT_1166792, C8R40DRAFT_1068383) was further intensified in the late storage stage (T2/T0 comparison), where the expression decreased substantially. However, in the T2/T0 and T2/T1 comparisons, a significant recovery was observed for certain laccase genes (C8R40DRAFT_642440), suggesting that different laccase family members may perform distinct cell wall modification tasks at various postharvest periods.

Based on the transcriptomic data, a series of DEGs associated with oxidative stress response and reactive oxygen species (ROS) scavenging were identified (Table 1). These DEGs mainly involve SOD, CAT, POD, and glutathione system-related enzymes. SOD family genes were predominantly up-regulated during storage. The manganese superoxide dismutase gene (C8R40DRAFT_1159243) was significantly up-regulated in the T1/T0 comparison group (log_2_FC = 1.02). In contrast, the Manganese/Iron-SOD gene (Mn/Fe-SOD, C8R40DRAFT_1163751) exhibited a significant down-regulation trend (T1/T0, log_2_FC = −1.13; T2/T0 comparison, log_2_FC = −1.20). On the other hand, the catalase gene (C8R40DRAFT_1058359) showed a consistent up-regulation trend in the T2/T0 comparison (log_2_FC = 1.30). Regarding peroxidases, the expression of manganese peroxidase was more complex. Gene C8R40DRAFT_1176895 was significantly down-regulated in both comparison groups, whereas gene C8R40DRAFT_328673 was significantly up-regulated in the early stage (log_2_FC = 2.74) but showed a down-regulation trend in the T2/T1 comparison (log_2_FC = −1.09).

### 3.5. Partial Least Squares Regression (PLSR) Analysis

To further elucidate the correlation between cell wall polysaccharide contents, cell wall metabolic enzyme activities, oxidative stress-related enzyme activities, and corresponding encoding genes’ transcriptome data of *L. edodes* during storage, a PLSR model was constructed using 18 key genes (Table 1) as predictor variables and 12 physiological indicators as response variables. The model demonstrated high explanatory power and predictive reliability, with cumulative explained variances of R^2^X = 0.762 and R^2^Y = 0.850. The cross-validation predictive index (Q^2^) reached 0.786 (Q^2^ > 0.5), indicating that the transcriptomic data effectively predicted the physiological changes during storage. Based on the Variable Importance in Projection (VIP) criteria, nine key regulatory genes were identified as significant contributors to the model (VIP > 1.0, Table S2). The genes with the VIP values greater than 1.0 were: 2 beta-glucan (C8R40DRAFT_775537, VIP = 1.46), laccase (C8R40DRAFT_642440, VIP = 1.26), catalase (C8R40DRAFT_1058359, VIP = 1.25), laccase (C8R40DRAFT_1166792, VIP = 1.22), manganese peroxidase (C8R40DRAFT_1176895, VIP = 1.21), manganese/iron superoxide dismutase (C8R40DRAFT_1163751, VIP = 1.20), 2 beta-glucanase (C8R40DRAFT_774955, VIP = 1.17), cellulase CEL6B (C8R40DRAFT_1075571, VIP = 1.16) and manganese superoxide dismutase (C8R40DRAFT_1159243, VIP = 1.16). These key genes are mainly involved in the cell wall metabolism and oxidative stress response processes of *L. edodes*, suggesting that they may be the core molecular targets regulating the changes in cell wall metabolism of *L. edodes* during storage.

## 4. Discussion

The short postharvest shelf life of *L. edodes* severely constrains its commercial circulation. Genomic and transcriptomic surveys have highlighted that the transition from harvest to storage triggers a massive reprogramming of the gene expression landscape in this species [17]. Crucially, alterations in cell wall metabolism are recognized as early biological events that precede detectable changes in macroscopic quality, such as texture softening [6,11]. While the eventual decline in firmness is a hallmark of senescence, it is the preceding enzymatic remodeling of cell wall polysaccharides that dictates the integrity of the fungal hyphae [12,18]. As recently underscored by Ost et al. [19], the fungal cell wall is a dynamic and complex structure whose regulation poses a significant challenge for postharvest biotechnology. Specifically, the degradation of core structural components such as lentinan has been linked to the targeted downregulation of specific cell wall metabolism enzymes during preservation [20]. Although previous studies have documented correlations between these hydrolytic enzyme activities and postharvest decay [13], the precise transcriptional architecture governing the early initiation of cell wall metabolism remains poorly understood. To bridge this gap, this study employed PLSR to mathematically isolate the most influential biological variables linking the transcriptome to early metabolic shifts. A core regulatory module comprising nine key genes with Variable Importance in Projection (VIP) scores > 1.0 was identified. The identification of these high-contribution targets provides novel molecular evidence for the initiation of cell wall remodeling, shifting the scientific focus from late-stage quality loss to the fundamental gene-level regulation of early cell wall metabolism.

### 4.1. Cell Wall Metabolism and Structural Disassembly

The structural integrity of the cell wall is the primary determinant of fungal firmness. The PLSR model indicated that gene variables related to cell wall metabolism, particularly β-glucanases and cellulases, exerted the strongest contribution to the physiological shifts in *L. edodes*. Specifically, the continuous decline in cellulose content was negatively correlated with the upregulation of Cellulase CEL6B (C8R40DRAFT_1075571, VIP = 1.16). In this dataset, the transcriptional upregulation of CEL6B (Log_2_FC = 1.45 in the T2/T0 comparison) temporally coincided with both the reduction in cellulose content and the peak of cellulase activity. CEL6B, a key cellobiohydrolase, exhibited sustained high expression in our dataset. Li et al. [18] reported similar transcriptomic changes and provided evidence linking elevated CEL6B expression to increased hydrolysis of crystalline cellulose, a process implicated in the loss of mechanical strength during storage. The fungal cell wall is a dynamic fiber–matrix network whose temperature- and time-dependent remodeling presents a major challenge for postharvest preservation [21]. The present results are consistent with those reports, showing coordinated upregulation of cell-wall hydrolase transcripts alongside increased enzymatic activities, which together suggest enhanced polymer disassembly under storage.

Interestingly, the contents of chitin and β-glucan exhibited an anomalous upward trend at certain time points. According to the latest structural insights from Singla et al. [22], β-glucan levels are highly dependent on their extraction state and molecular weight. Thus, the apparent increase observed here should be interpreted as enzymatic “remodelling” rather than structural reinforcement. High-VIP β-glucanases (C8R40DRAFT_775537, VIP = 1.46) and C8R40DRAFT_774955 (VIP = 1.17) likely function as “remodelling shears” that cleave covalent and non-covalent chitin-glucan cross-links, consistent with observations reported by Konno et al. [20]. This cleavage fragments high-molecular-weight polymers into soluble oligomers, elevating assayable soluble fractions while disrupting matrix integrity, which is a core structural challenge identified in filamentous fungi, and thereby weakening cell-wall rigidity at the microscopic level [21]. Consequently, the transcriptional activation of CEL6B and specific β-glucanases represents a major execution unit of cell wall disassembly. This process sets the stage for investigating the putative role of the laccase-ROS-transcriptional factor module in regulating senescence, where oxidative stress may indirectly promote cell wall weakening prior to or alongside enzymatic degradation. Furthermore, it should be noted that certain apparent variations in measured cell wall polysaccharide contents may also reflect changes in the extractability or detectability of specific cell wall fractions during storage, rather than absolute increases in their biosynthesis.

The concurrent increase in measured chitin content and chitinase activity warrants caution in interpretation. An apparent rise in chitin as assayed here does not necessarily indicate net biosynthetic accumulation of high-molecular-weight polymer. Changes in polymer extractability, solubility, or fragmentation can increase the assay-detectable fraction without net synthesis. Upregulation of chitinase expression and activity may represent an early or localized response to cell-wall remodeling that has not yet produced measurable net loss of bulk polymer, or may be counterbalanced by simultaneous modifications such as re-crosslinking or deposition in other wall compartments. In addition, enzymatic activity measured in crude extracts reflects potential activity under assay conditions and may not directly translate into in situ degradation if substrate accessibility or local inhibitors differ. For these reasons, the observed patterns are best interpreted as indicative of active cell-wall remodeling dynamics rather than simple net degradation.

### 4.2. ROS Metabolism and Oxidative Stress Drive Cell Wall Disassembly

While enzymatic hydrolysis constitutes the execution machinery of cell wall disassembly, the upstream trigger lies in the disruption of cellular redox homeostasis. The physiological data revealed a surge in MDA content and LOX activity during late storage, indicating severe membrane lipid peroxidation. This loss of membrane integrity inevitably compromises subcellular compartmentalization, facilitating the leakage of hydrolytic enzymes and their subsequent interaction with cell wall substrates [17,23]. PLSR analysis further corroborated this link by identifying catalase (C8R40DRAFT_1058359, VIP = 1.25) and two SODs as critical variables covarying with textural decay, suggesting a tight coupling between the antioxidant system and cell wall metabolism.

The divergent response patterns of high-VIP SOD genes reveal a spatiotemporal heterogeneity in ROS scavenging. The upregulation of Mn-SOD (C8R40DRAFT_1159243, VIP = 1.16) likely represents an active attempt to scavenge mitochondrial superoxide anions, whereas the downregulation of Mn/Fe-SOD (C8R40DRAFT_1163751, VIP = 1.20) implies a failure in cytosolic or specific organelle ROS clearance. This divergent expression alters the composition and spatiotemporal heterogeneity of the ROS pool across different cellular compartments, creating localized peaks or defects of ROS. This likely promotes localized cell wall oxidation and selective degradation, potentially explaining the non-uniform remodeling observed within the tissue [24].

Catalase (C8R40DRAFT_1058359, VIP = 1.25) exhibited a sharp upregulation at the T2 stage, signifying a “lagged compensatory response” to the H_2_O_2_ burst. Biologically, CAT both protects membrane systems from H_2_O_2_-induced damage and regulates H_2_O_2_ when it functions as a signaling molecule [25]. Thus, its upregulation might partially alleviate oxidative damage while simultaneously dampening H_2_O_2_ signal intensity, potentially delaying or suppressing the transcription/activity of specific hydrolases that rely on H_2_O_2_ signaling. However, in this study, the compensatory upregulation of CAT failed to prevent senescence, suggesting that the rate of ROS generation or local ROS dynamics exceeded the buffering capacity of the cellular antioxidant system [25].

Furthermore, the significant downregulation of Manganese Peroxidase (MnP, C8R40DRAFT_1176895, VIP = 1.21) suggests a compromised capacity of *L. edodes* to utilize H_2_O_2_ for modifying lignin or phenolic polymers in the late storage period. Since MnP, alongside laccase, functions in regulating the phenolic matrix and cross-links of the cell wall, its downregulation implies a loss of the fungal capacity to “remodel” phenolic cross-links via oxidative pathways [26]. This indirectly alters the chemical environment of the cell wall and may facilitate non-enzymatic oxidative damage to the polysaccharide network. In conclusion, ROS accumulation drives cell wall remodeling through a synergistic mechanism involving direct non-enzymatic oxidative cleavage of polysaccharide chains and the oxidative stress-induced transcriptional reprogramming of cell wall-degrading enzymes [24,27,28]. The observations support the hypothesis that oxidative stress may facilitate polysaccharide depolymerization—either indirectly by altering the transcriptional regulation of hydrolases or by promoting chemical modifications that increase substrate accessibility to enzymes—however, direct non-enzymatic cleavage of polysaccharides by ROS was not demonstrated here and requires targeted biochemical validation.

### 4.3. Laccase Act as Regulatory Hubs Linking Oxidative Stress to Wall Disassembly

The identification of Laccases (C8R40DRAFT_642440, VIP = 1.26; C8R40DRAFT_1166792, VIP = 1.22) as high-impact variables in the PLSR model points to a sophisticated regulatory mechanism beyond simple hydrolysis. Laccases perform a dual role in fungi by catalyzing the oxidation of phenolic substrates that can lead to inter-molecular cross-linking and polymerization (e.g., modification of lignin/phenolics), thereby modulating the chemical cross-linking state and mechanical properties of the cell wall. Simultaneously, they mediate browning reactions and contribute to environmental-response pathways, acting as key effectors of oxidative stress responses [29,30,31]. Consequently, the integrity of the phenolic barrier, defined by the density of cross-links that shield polysaccharides from enzymatic hydrolysis, emerges as a principal determinant of *L. edodes* texture, primarily governed by laccase activity.

A functional division of labor within the laccase family is proposed, with certain members prioritizing early phenolic cross-linking and stabilization to maintain cell wall integrity while others predominantly mediate oxidative modification during later stages or under elevated ROS conditions [32]. Distinct expression dynamics of high-VIP laccase genes support a model in which upstream ROS signals are translated into downstream structural failure, such that downregulation or fluctuation of specific laccase members, notably C8R40DRAFT_1166792, interrupts maintenance of phenolic cross-links between cell wall polymers, loosens matrix porosity, and increases substrate accessibility for hydrolases such as CEL6B and β-glucanases [33,34]. Although the systems differ, the principle of “phenolic-mediated cross-linking as a physical/chemical barrier to polysaccharides” is a widespread biochemical tenet. Research by Franco Cairo et al. [27] demonstrates that in non-plant systems, oxidative cleavage similarly boosts glycoside hydrolase accessibility, providing a trans-systemic experimental bridge for extrapolating these mechanisms to the fungal cell wall. Simultaneously, manganese peroxidase (MnP), as an H_2_O_2_-dependent enzyme, serves as a direct coupling node between the antioxidant system and the cell wall by working in concert with laccases to remodel the phenolic matrix [26,35]. The significant downregulation of MnP (VIP = 1.21) coincides with the disruption of H_2_O_2_ homeostasis, suggesting that the fungus loses its ability to finely tune cell wall rheology via peroxidase-mediated remodeling, leaving the wall vulnerable to unregulated oxidative damage.

**Figure 4:**
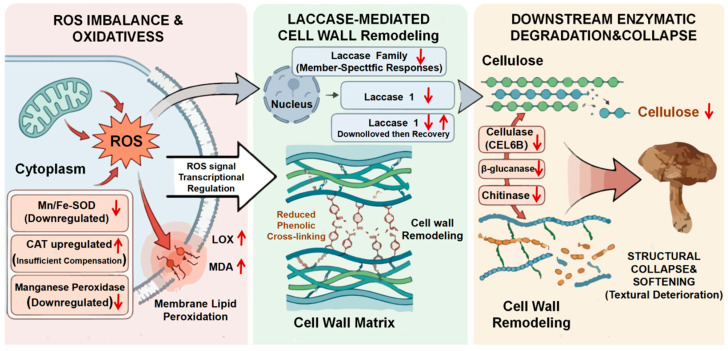
Proposed laccase-ROS-hydrolase regulatory network governing postharvest cell wall remodeling. Upward arrows represent gene expression up-regulation and downward arrows represent down-regulation while two arrows after the *Laccase 1* gene indicate its expression is first up-regulated then down-regulated.

Consequently, we propose a putative regulatory network linking ROS, laccase family members, and hydrolases that may underlie postharvest wall remodeling (Figure 4). The physiological and transcriptomic data suggest that an upstream imbalance in ROS homeostasis—driven by the suppression of Mn/Fe-SOD and the insufficiency of CAT—creates a pervasive oxidative environment [36]. This environment likely alters the transcriptional regulation of specific laccase genes, potentially mediated by ROS-sensitive transcription factors [37,38]. Such regulatory shifts may modulate phenolic cross-linking, thereby indirectly influencing the accessibility of polysaccharide substrates to hydrolases. This integrated network represents a conceptual model derived from association analyses and requires further experimental validation to establish causality. Acting as a midstream gateway, the subsequent dysregulation of laccase activity, supplemented by MnP, diminishes the density of phenolic cross-links. This chemical alteration effectively unlocks the cell wall matrix, dismantling the protective physical barrier that typically restricts enzyme penetration [33,34]. This cascade culminates in the downstream execution phase, where the loosened carbohydrate matrix becomes highly accessible to the up-regulated cellulase and β-glucanases, leading to the rapid and irreversible depolymerization of the structural network [18]. Concurrently, the redirection of any remaining laccase activity towards free phenolic substrates, in conjunction with PPO, accelerates the browning reactions that are phenotypically synchronized with tissue softening [39,40].

Several limitations should be acknowledged. The relationships identified among ROS dynamics, laccase activity, and cell wall remodeling are primarily based on correlative evidence derived from physiological measurements, enzyme activity assays, transcriptomic analysis, and multivariate statistical modeling. Direct biochemical evidence demonstrating ROS-mediated cleavage of cell wall polysaccharide chains was not obtained. In addition, functional validation of the candidate regulatory genes and enzymes involved in the proposed regulatory framework was not performed. Therefore, the mechanistic relationships discussed in this study should be interpreted as plausible associations rather than experimentally confirmed causal interactions.

Our findings, while primarily correlative, point to several practical strategies that could help preserve shiitake quality by modulating the upstream factors we profiled (ROS dynamics, laccase activity, and cell-wall-remodeling enzymes). First, strict control of cold storage conditions (low temperature, high relative humidity, and optimized O_2_/CO_2_ atmospheres) can reduce respiration-driven ROS production and slow cell-wall degradation, as shown for shiitake under modified-atmosphere storage. Such storage control may therefore help maintain firmness and delay cell-wall polysaccharide loss [41]. Second, surface treatments and edible coatings (e.g., chitosan-based films, coatings enriched with antioxidant or antimicrobial natural extracts) can reduce water loss, limit enzymatic browning, and lower oxidative damage, and thus represent practical, industry-feasible interventions to prolong shelf life [42,43]. Third, considering the central role of fungal laccases as multicopper oxidases capable of catalyzing the oxidation and polymerization of phenolic compounds, strategies that regulate laccase activity or its phenolic substrates may also influence postharvest quality changes. Previous studies have shown that laccase expression and activity in mushrooms are associated with postharvest physiological processes, including browning reactions and the regulation of phenolic metabolism. Therefore, monitoring or modulating laccase-related pathways may represent a potential target for future preservation strategies and quality evaluation [44,45].

## 5. Conclusions

In conclusion, this study provides a comprehensive mechanistic model for the postharvest remodeling of *L. edodes*, demonstrating that this process is a sophisticated biological cascade triggered by oxidative stress and executed through the synergistic action of laccases and cell wall degrading enzymes Our findings reveal that the initiation of senescence stems from the disruption of ROS homeostasis, where the regulatory imbalance of antioxidant guardians characterized by the downregulation of Mn/Fe-SOD and the insufficient compensatory response of CAT leads to pervasive membrane lipid peroxidation. This redox shift is associated with transcriptional changes in laccases and hydrolases and with biochemical indicators of oxidative stress. These observations are consistent with a hypothesis that altered laccase activity and oxidative modification of phenolic components may reduce phenolic cross-linking, which in turn could increase accessibility of structural polysaccharides to hydrolases. However, the causal sequence leading to irreversible structural collapse remains to be functionally established. Although the correlation-based evidence from the PLSR model is robust, future functional studies, such as heterologous expression or gene silencing, are required to definitively confirm the individual roles of these candidate genes. Overall, this research advances the understanding of fungal postharvest physiology from descriptive quality observations to a gene-level mechanistic framework, providing a theoretical foundation for extending the commercial shelf life of edible mushrooms.

## Figures and Tables

**Table 1:** Gene expression of DEGs related to cell wall metabolism and oxidative stress.

GeneID	Products	log_2_FC		
		T1/T0	T2/T0	T2/T1
C8R40DRAFT_775537	2 beta-glucan	1.82	1.9	-
C8R40DRAFT_774955	2 beta-glucanase	1.26	4.57	3.31
C8R40DRAFT_1102963	beta-D-xylosidase/beta-D-glucosidase	1.02	-	-
C8R40DRAFT_1103272	beta-D-xylosidase/beta-D-glucosidase	-	-	−1.03
C8R40DRAFT_1055999	beta-glucan synthesis-associated	1.03	-	-
C8R40DRAFT_1075571	cellulase CEL6B	1.3	1.45	-
C8R40DRAFT_1127624	cellulase CEL7A	1.03	-	-
C8R40DRAFT_229556	chitin synthase-domain-containing protein	−1.14	-	-
C8R40DRAFT_1058826	exo-beta-1,3-glucanase	2.99	2.36	-
C8R40DRAFT_1173381	glucan endo-1,6-beta-glucosidase	4.29	2.15	−2.14
C8R40DRAFT_1068383	laccase	−1.51	−1.11	-
C8R40DRAFT_1166792	laccase	-	−1.16	-
C8R40DRAFT_642440	laccase	−1.35	1.12	2.47
C8R40DRAFT_1177832	laccase 1	1.58	-	-
C8R40DRAFT_1171887	putative chitinase	−1.48	-	1.43
C8R40DRAFT_1058359	catalase	1.29	1.3	-
C8R40DRAFT_1176895	manganese peroxidase	−2.17	−1.84	-
C8R40DRAFT_328673	manganese peroxidase	2.74	-	−1.09
C8R40DRAFT_1159243	manganese superoxide dismutase	1.02	-	-
C8R40DRAFT_1163751	manganese/iron superoxide dismutase	−1.13	−1.2	-

“-” indicated gene was not differentially expressed.

## Data Availability

The original contributions presented in this study are included in the article/Supplementary Material. Further inquiries can be directed to the corresponding authors.

## Supplementary Materials

The following supporting information can be downloaded at https://www.mdpi.com/article/10.3390/foods15061039/s1, Figure S1: The principal component analysis (PCA) and hierarchical cluster analysis of DEGs. Sample labels: T0 = Day 0, T1 = Day 4, T2 = Day 8; Figure S2: Number of differentially expressed genes (DEGs) and corresponding volcano plots. (A) number of DEGs, (B) volcano plots of T1/T0 comparison group, (C) volcano plots of T2/T0 comparison group, (D) volcano plots of T2/T1 comparison group. Sample labels: T0 = Day 0, T1 = Day 4, T2 = Day 8; Figure S3: GO and KEGG enrichment bubble plot of DEGs. (A) GO enrichment of T1/T0 comparison group, (B) GO enrichment of T2/T0 comparison group, (C) GO enrichment of T2/T1 comparison group, (D) KEGG enrichment of T1/T0 comparison group, (E) KEGG enrichment of T2/T0 comparison group, (F) KEGG enrichment of T2/T1 comparison group. T0 = Day 0, T1 = Day 4, T2 = Day 8; Table S1: The differentially expressed genes (DEGs) of *Lentinula edodes* in different storage times; Table S2: Variable Importance in Projection (VIP) criteria of gene related to cell wall metabolism and oxidative stress.
