# Supplementary material for: Transcriptome Analysis of Postharvest Lentinula edodes Cell Wall Metabolism During Storage Indicating a Laccase-Mediated Regulatory Network
Source: Foods. 2026 Mar 16;15(6):1039. doi: 10.3390/foods15061039 (PMC13025948; doi:10.3390/foods15061039)
Supplement: Supplementary file 1 [file foods-15-01039-s001.zip › Supplementary figures.pdf]

Supplementary figures

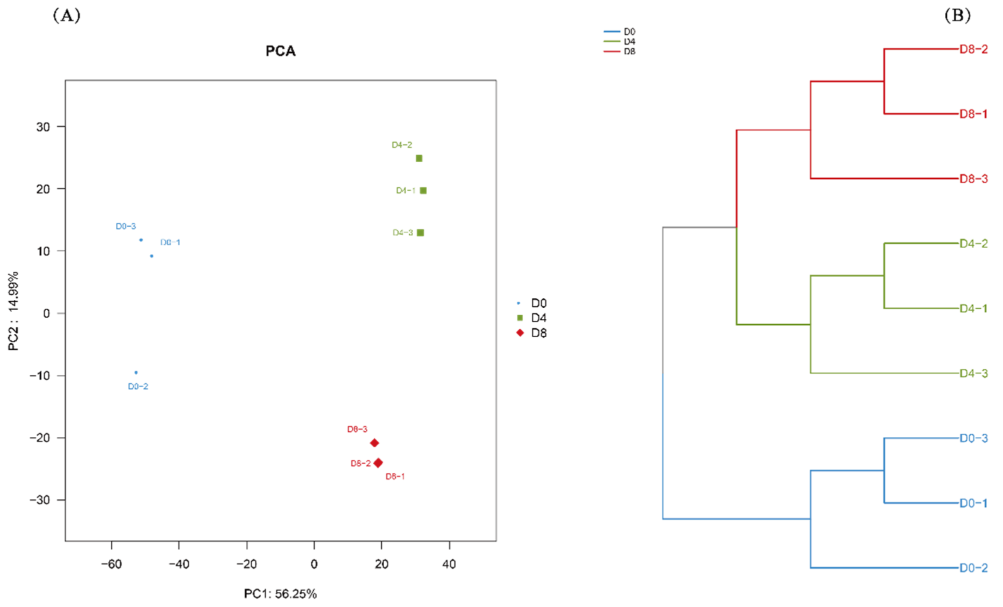

Figure S1 The principal component analysis (PCA) and hierarchical cluster analysis of DEGs.

Sample labels: T0 = Day 0, T1 = Day 4, T2 = Day 8.

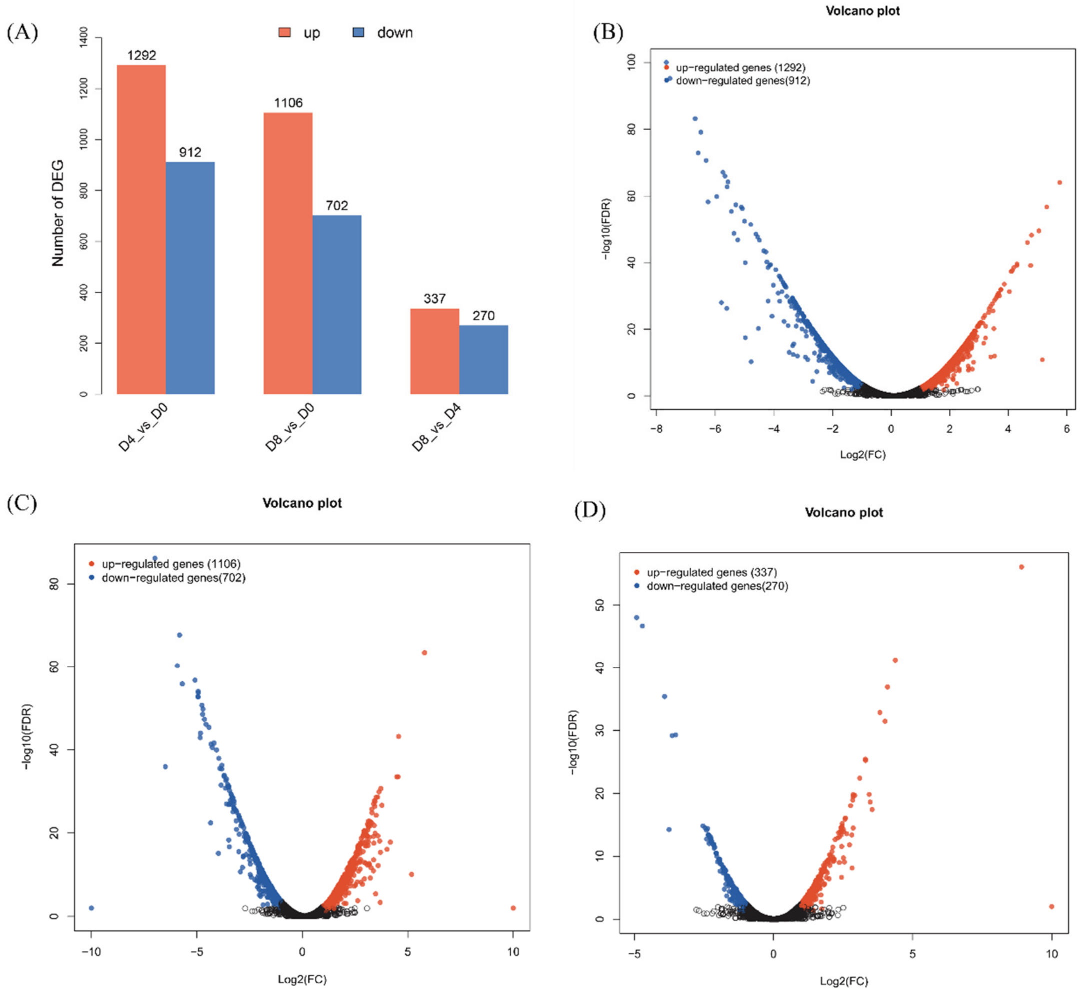

Figure S2 Number of deferentially expressed genes (DEGs) and corresponding volcano plots. (A) number of DEGs, (B) volcano plots of T1/T0 comparison group, (C) volcano plots of T2/T0 comparison group, (D) volcano plots of T2/T1 comparison group. Sample labels: T0 = Day 0, T1 = Day 4, T2 = Day 8.

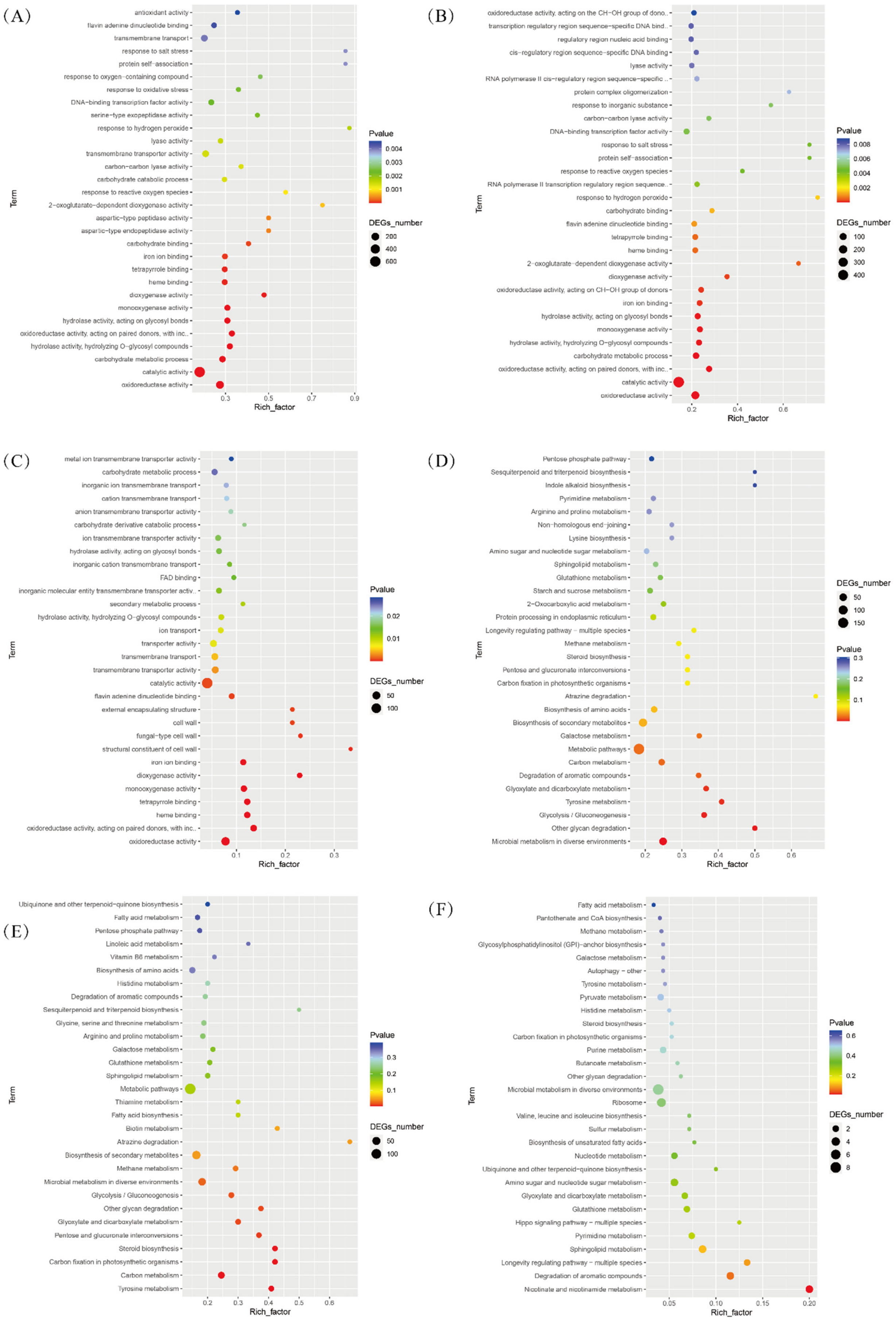
(A)
antioxidant activity
flavin adenine dinucleotide binding
transmembrane transport
response to salt stress
protein self-association
response to oxygen-containing compound
response to oxidative stress
DNA-binding transcription factor activity
serine-type exopeptidase activity
response to hydrogen peroxide
lyase activity
transmembrane transporter activity
carbon-carbon lyase activity
carbohydrate catabolic process
response to reactive oxygen species
2-oxoglutarate-dependent dioxygenase activity
aspartic-type peptidase activity
aspartic-type endopeptidase activity
carbohydrate binding
iron ion binding
tetrapyrrole binding
heme binding
dioxygenase activity
monooxygenase activity
hydrolase activity, acting on glycosyl bonds
oxidoreductase activity, acting on paired donors, with inc..
hydrolase activity, hydrolyzing O-glycosyl compounds
carbohydrate metabolic process
catalytic activity
oxidoreductase activity
Term
Rich_factor
Pvalue
DEGs_number
(B)
oxidoreductase activity, acting on the CH-OH group of dono..
transcription regulatory region sequence-specific DNA bind..
regulatory region nucleic acid binding
cis-regulatory region sequence-specific DNA binding
lyase activity
RNA polymerase II cis-regulatory region sequence-specific ..
protein complex oligomerization
response to inorganic substance
carbon-carbon lyase activity
DNA-binding transcription factor activity
response to salt stress
protein self-association
response to reactive oxygen species
RNA polymerase II transcription regulatory region sequence..
response to hydrogen peroxide
carbohydrate binding
flavin adenine dinucleotide binding
tetrapyrrole binding
heme binding
2-oxoglutarate-dependent dioxygenase activity
dioxygenase activity
oxidoreductase activity, acting on CH-OH group of donors
iron ion binding
hydrolase activity, acting on glycosyl bonds
monooxygenase activity
hydrolase activity, hydrolyzing O-glycosyl compounds
carbohydrate metabolic process
oxidoreductase activity, acting on paired donors, with inc..
catalytic activity
oxidoreductase activity
Term
Rich_factor
Pvalue
DEGs_number
(C)
metal ion transmembrane transporter activity
carbohydrate metabolic process
inorganic ion transmembrane transport
cation transmembrane transport
anion transmembrane transporter activity
carbohydrate derivative catabolic process
ion transmembrane transporter activity
hydrolase activity, acting on glycosyl bonds
inorganic cation transmembrane transport
FAD binding
inorganic molecular entity transmembrane transporter activ..
secondary metabolic process
hydrolase activity, hydrolyzing O-glycosyl compounds
ion transport
transporter activity
transmembrane transport
transmembrane transporter activity
catalytic activity
flavin adenine dinucleotide binding
external encapsulating structure
cell wall
fungal-type cell wall
structural constituent of cell wall
iron ion binding
dioxygenase activity
monooxygenase activity
tetrapyrrole binding
heme binding
oxidoreductase activity, acting on paired donors, with inc..
oxidoreductase activity
Term
Rich_factor
Pvalue
DEGs_number
(D)
Pentose phosphate pathway
Sesquiterpenoid and triterpenoid biosynthesis
Indole alkaloid biosynthesis
Pyrimidine metabolism
Arginine and proline metabolism
Non-homologous end-joining
Lysine biosynthesis
Amino sugar and nucleotide sugar metabolism
Sphingolipid metabolism
Glutathione metabolism
Starch and sucrose metabolism
2-Oxocarboxylic acid metabolism
Protein processing in endoplasmic reticulum
Longevity regulating pathway - multiple species
Methane metabolism
Steroid biosynthesis
Pentose and glucuronate interconversions
Carbon fixation in photosynthetic organisms
Atrazine degradation
Biosynthesis of amino acids
Biosynthesis of secondary metabolites
Galactose metabolism
Metabolic pathways
Carbon metabolism
Degradation of aromatic compounds
Glyoxylate and dicarboxylate metabolism
Tyrosine metabolism
Glycolysis / Gluconeogenesis
Other glycan degradation
Microbial metabolism in diverse environments
Term
Rich_factor
DEGs_number
Pvalue
(E)
Ubiquinone and other terpenoid-quinone biosynthesis
Fatty acid metabolism
Pentose phosphate pathway
Linoleic acid metabolism
Vitamin B6 metabolism
Biosynthesis of amino acids
Histidine metabolism
Degradation of aromatic compounds
Sesquiterpenoid and triterpenoid biosynthesis
Glycine, serine and threonine metabolism
Arginine and proline metabolism
Galactose metabolism
Glutathione metabolism
Sphingolipid metabolism
Metabolic pathways
Thiamine metabolism
Fatty acid biosynthesis
Biotin metabolism
Atrazine degradation
Biosynthesis of secondary metabolites
Methane metabolism
Microbial metabolism in diverse environments
Glycolysis / Gluconeogenesis
Other glycan degradation
Glyoxylate and dicarboxylate metabolism
Pentose and glucuronate interconversions
Steroid biosynthesis
Carbon fixation in photosynthetic organisms
Carbon metabolism
Tyrosine metabolism
Term
Rich_factor
Pvalue
DEGs_number
(F)
Fatty acid metabolism
Pantothenate and CoA biosynthesis
Methane metabolism
Glycosylphosphatidylinositol (GPI)-anchor biosynthesis
Galactose metabolism
Autophagy - other
Tyrosine metabolism
Pyruvate metabolism
Histidine metabolism
Steroid biosynthesis
Carbon fixation in photosynthetic organisms
Purine metabolism
Butanoate metabolism
Other glycan degradation
Microbial metabolism in diverse environments
Ribosome
Valine, leucine and isoleucine biosynthesis
Sulfur metabolism
Biosynthesis of unsaturated fatty acids
Nucleotide metabolism
Ubiquinone and other terpenoid-quinone biosynthesis
Amino sugar and nucleotide sugar metabolism
Glyoxylate and dicarboxylate metabolism
Glutathione metabolism
Hippo signaling pathway - multiple species
Pyrimidine metabolism
Sphingolipid metabolism
Longevity regulating pathway - multiple species
Degradation of aromatic compounds
Nicotinate and nicotinamide metabolism
Term
Rich_factor
Pvalue
DEGs_number

Figure S3 GO and KEGG enrichment bubble plot of DEGs. (A) GO enrichment of T1/T0 comparison group, (B) GO enrichment of T2/T0 comparison group, (C) GO enrichment of T2/T1 comparison group, (D) KEGG enrichment of T1/T0 comparison group, (E) KEGG enrichment of T2/T0 comparison group, (F) KEGG enrichment of T2/T1 comparison group.

T0 = Day 0, T1 = Day 4, T2 = Day 8.
