# Supplementary material for: Transcriptome Analysis of Postharvest Lentinula edodes Cell Wall Metabolism During Storage Indicating a Laccase-Mediated Regulatory Network
Source: Foods. 2026 Mar 16;15(6):1039. doi: 10.3390/foods15061039 (PMC13025948; doi:10.3390/foods15061039)
Supplement: Supplementary file 1 [file foods-15-01039-s001.zip › Table S2.pdf]

Table S2. Variable Importance in Projection (VIP) criteria of gene related to cell wall metabolism and oxidative stress

GeneID	Products	VIP Value
C8R40DRAFT_1058826	exo-beta-1,3-glucanase	1.968
C8R40DRAFT_774955	2 beta-glucanase	1.863
C8R40DRAFT_1058359	catalase	1.736
C8R40DRAFT_1127624	cellulase CEL7A	1.642
C8R40DRAFT_1166792	laccase	1.529
C8R40DRAFT_1159243	manganese superoxide dismutase	1.412
C8R40DRAFT_1075571	cellulase CEL6B	1.245
C8R40DRAFT_1173381	glucan endo-1,6-beta-glucosidase	1.186
C8R40DRAFT_1102963	beta-D-xylosidase/beta-D-glucosidase	1.032
C8R40DRAFT_642440	laccase	0.934
C8R40DRAFT_775537	2 beta-glucan	0.924
C8R40DRAFT_1163751	manganese/iron superoxide dismutase	0.897
C8R40DRAFT_1103272	beta-D-xylosidase/beta-D-glucosidase	0.841
C8R40DRAFT_1177832	laccase 1	0.796
C8R40DRAFT_1068383	laccase	0.712
C8R40DRAFT_328673	manganese peroxidase	0.643
C8R40DRAFT_229556	chitin synthase-domain-containing protein	0.673
C8R40DRAFT_1176895	manganese peroxidase	0.527
